# Exchange of a Tracheal Tube and Supraglottic Airway Device: Evaluation of Different Techniques in Three Simulated Airway Scenarios (TUBE Study)—A Prospective, Randomised Controlled Study

**DOI:** 10.3390/jcm13010016

**Published:** 2023-12-19

**Authors:** Marc Kriege, Tim Demare, Robert Ruemmler, Irene Schmidtmann, Janosh Wojciechowski, Anneke Busch, Thomas Ott

**Affiliations:** 1 Department of Anaesthesiology, University Medical Centre, Johannes Gutenberg—University Mainz, 55131 Mainz, Germany; 2 Institute for Medical Biostatistics, Epidemiology and Informatics, University Medical Centre, Johannes Gutenberg-University Mainz, 55131 Mainz, Germany; 3 Department of Anaesthesiology and Intensive Care, Asklepios Paulinen Hospital Wiesbaden, 65197 Wiesbaden, Germany

**Keywords:** airway management, existing airway device, tracheal intubation, simulation, scenario training, anaesthesiology, laryngoscopy, laryngeal masks

## Abstract

Background: The swapping of a supraglottic airway device or a tracheal tube in anaesthetised adult patients is a challenging procedure because potential complications through hypoxemia and loss of airway may occur, with life-threatening implications. This study aims to evaluate which airway technique offers the highest success rate concerning a secure airway in established supraglottic airway and tracheal tube airway exchange scenarios. Methods: After ethical approval, anaesthesiologists were randomised 1:1 into simulated scenarios: an LTS group (malpositioned laryngeal tube) and a Cuff group (relevant cuff leakage of a placed tracheal tube). After that, both groups completed a common scenario consisting of a partially obstructed tracheal tube lumen in a fixed prone position with a Mayfield clamp. The primary endpoint was a successful tracheal airway exchange within ten minutes after the start of the scenario and before severe hypoxemia (SpO_2_ < 80%) arose. Secondary endpoints were the evaluation of factors influencing success after 10 min. Results: In total, 60 anaesthesiologists (LTS group *n* = 30; Cuff group *n* = 30) with a median experience of 7 years (IQR 4-11) were observed. Within 10 min, a malpositioned laryngeal tube was successfully exchanged by 27/30 (90%) participants, compared to the exchange of a tracheal tube with a relevant cuff leakage by 29/30 (97%; *p* > 0.05). An airway exchange in an obstructed tube scenario occurred in 22/59 (37%). Loss of airway maintenance showed an obvious association with failure in the common scenario (*p* = 0.02). Conclusion: The results of this simulation-based study reflect that the exchange of an existing but insufficient airway device in clinical practice is a high-risk procedure. Especially in a fixed prone position, the deliberate evaluation of the existing airway patency and well-conceived airway management in the case of the accidental loss of the airway or obstructed airway access are crucial.

## 1. Introduction

The exchange of existing airway management with a tracheal tube (TT) or a supraglottic airway device (SGA) in elective surgery patients or critical care patients is a simple concept but not a simple intervention. The loss of the airway and severe hypoxemia may occur, with life-threatening ramifications [1,2,3,4,5]. A TT or SGA may require urgent replacement under the following conditions: if the TT is malpositioned, or blocked/obstructed, if the cuff has a leakage, an SGA needs to be upgraded to a TT, a single TT needs to be changed to a double-lumen tube, or if a small TT has been inserted during difficult or flexible endoscopic intubation. Recent data show a relevant incidence of between 11% and 64% of patients who need to receive a change of out-of-hospital airway management during emergency care due to insufficient ventilation [2,4]. Numerous case reports and several studies have been published concerning complications or successfully applied techniques in such situations [6,7,8,9,10,11,12]. Limited visualisation because of the established TT, secretions, oedema in patients with sepsis, limited spine mobility due to cervical spine injury, and restricted positioning resources may complicate laryngoscopy, especially conventional direct laryngoscopy (DL) [10]. Furthermore, physicians should be aware of the risk of tongue swelling, which may be related to either prolonged placement, ignoring the high cuff pressure of the SGA or excessive balloon volumes of the laryngeal tube [13].

There are many methods that can be used for TT or SGA exchange to reduce severe adverse events during the procedure. All safe methods should include optimal glottis visualisation, clearing the pathway for TT exchange, and ensuring airway continuity with airway exchange catheters (AECs) [11,12,14]. Recently, the use of video laryngoscopy (VL) has increased glottis visualisation and intubation success in difficult airway situations [14,15,16,17,18,19]. To ensure airway continuity during TT or SGA exchange, intubating catheters or AECs are available and can be used as a single method for TT exchange or guided intubation by a flexible endoscope through an SGA [12]. Furthermore, improved visualisation during the AEC-assisted TT exchange has resulted in a first-pass success rate of 96% [16], and VL served as its own rescue device when the accidental removal of the AEC occurred during the exchange. Unintentional extubation or an obstructed lumen of the TT while the patient is in the prone position is a potentially life-threatening situation [20,21,22,23,24,25,26]. Recently, a case report and several studies recommended techniques and algorithms for rescue airway management in patients whose tracheas were unintentionally extubated during the course of general anaesthesia in the operating theatre [20,21,22,23]. Further important factors are common human factor themes that have been found to contribute to or cause significant errors that lead to patient morbidity and mortality in airway management [23,24,25]. Poor communication and teamwork, system errors with a failure to check, and individual failings such as fixation error, inadequate leadership, poor situation awareness, and fatigue are recurring themes [27].

In addition to case reports and observational studies, there is no publication systematically evaluating what physicians in charge really do to manage these airway complications in special circumstances. Due to the dynamics and imminent life-threatening events, as well as the unpredictable incidence of such situations, it is difficult to study this in clinical reality. Thus, we decided to design a controlled simulation-based study to evaluate techniques and airway devices applied successfully by anaesthesiologists. We chose the use of simulation for this study to collect data, and recruited anaesthesiologists with different levels of experience in a controlled and safe environment [28]. The aim of the study was to evaluate which airway technique offers the highest success rate concerning a secure airway in established SGA and TT airway exchange scenarios.

## 2. Methods

### 2.1. Ethical Considerations

The ethics committee of the Medical Association of the state of Rhineland Palatine (Germany) approved the study (Registration Nr.: 2019–14650; Chairperson Dr A Wagner) on 28 October 2019. This trial was conducted in adherence to the current version of the Declaration of Helsinki and the GCP guidelines. The trial was registered at ClinicalTrials.gov, Identifier: NCT04158271. Written informed consent was obtained from all participants.

### 2.2. Study Design

The study was performed in accordance with the CONSORT Statement [29]. This was a prospective, randomised, controlled study encompassing three different airway scenarios at a tertiary university hospital from 11 November to 15 November 2019, designed to evaluate the endpoints of the present study.

### 2.3. Study Setting

The study was conducted in the simulation centre of the Department of Anaesthesiology of the University Medical Centre Mainz, Germany. Each participant had to manage either a malpositioned laryngeal tube (LTS) scenario or relevant cuff leakage of a placed tracheal tube (CUFF) scenario first. The second scenario was a partially obstructed tracheal tube lumen in the fixed prone position with a Mayfield clamp (PRONE) for all participants, for educational reasons. The data were collected during the annual institutional simulation-based airway training in November 2019.

#### 2.3.1. Material

The airway of an adult high-fidelity simulator (SimMan™ 3G; Laerdal^®^ Medical, Stavanger, Norway) was manipulated according to the needs of the particular scenario by the manufacturer’s settings for tongue oedema and pharyngeal obstruction, whereas adequate chest excursion and breath sounds were adjusted according to the scenario. The departmental anaesthesia ventilator (Pallas, Dräger^®^, Lübeck, Germany), standardised airway, and anaesthesia trolley were used. The airway trolley included the following devices: (i) one type of videolaryngoscope (VL) C-MAC^®^ Macintosh blade, sizes 3 and 4, or hyperangulated blade (Karl Storz^®^, Tuttlingen, Germany), or standard laryngoscope, with a Macintosh blade, sizes 3 and 4 (DL; Heine^®^ Optotechnik, Gilching, Germany); (ii) a supraglottic airway device (SGA): Ambu^®^ Aura Gain^®^, sizes 4 and 5 (Ambu^®^ A/S, Copenhagen, Denmark); (iii) different airway exchange catheters (AECs): Cook^®^ Airway Exchange Catheter (Cook^®^ Medical, Bloomington, IN, USA), S-Guide™, or Muallem Stylet™ (VBM^®^, Sulz a. N., Germany), and Aintree Intubation Catheter™ (Cook^®^ Medical, Bloomington, IN, USA); and (iv) a flexible (video) endoscope (FO): aScope^®^, sizes slim to large (Ambu^®^ A/S, Copenhagen, Denmark).

#### 2.3.2. Scenarios and Sequence

**Figure 1:**
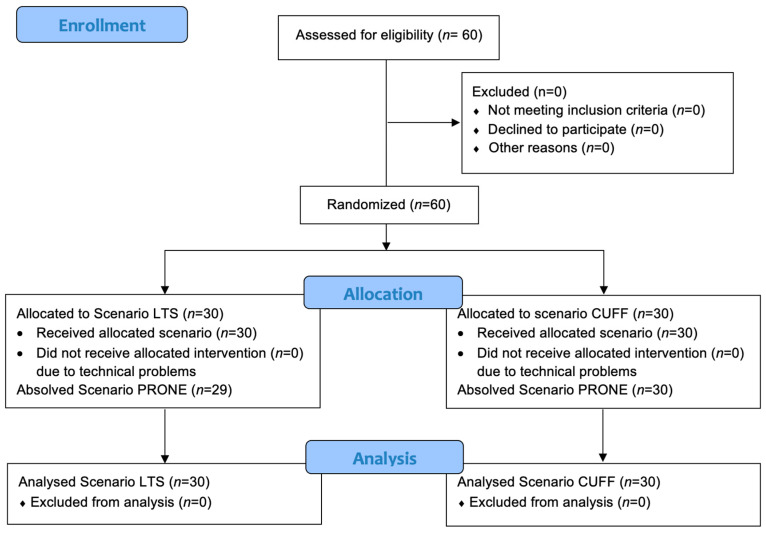
Consort diagram.

The participants were first randomly assigned to one of two scenarios, either the LTS group or the CUFF group. After that, all of them underwent the PRONE scenario (Figure 1). Randomisation concerning the first scenario was performed using GraphPad^®^ QuickCalcs; accessed on 1 September 2019; (http://www.graphpad.com) by generating a number defining a sequence to either one of the two scenarios. Neither the participating anaesthesiologist nor the simulation team were aware of what allocation was next in the randomisation sequence.

The scenarios were controlled by three instructors: two were technical supervisors, and one was assigned to complete one protocol for each scenario. An additional study nurse not involved in the simulation scenario was present during the study to record the study parameters.

(i) LTS scenario: A relevant malpositioning of the LTS with the necessity for LTS exchange for surgery. The risk factors concerning the airway were an upper airway oedema, tongue swelling, and pharyngeal oedema 3 h after the initial inflation of the cuffs. In these time frames, complications are evident in 39% of patients, whereas tongue engorgement is seen in 7% [6,13].

(ii) CUFF scenario: A relevant cuff leakage of the TT with the necessity tracheal tube exchange. Risk factors concerning the airway were a positive fluid balance of 3000 mL/48 h, with a laryngeal oedema [30].

(iii) PRONE scenario: An obstructed TT [20,23,24] causing insufficient oxygenation and ventilation during ongoing surgery. The risk factors concerning the airway were a fixed prone position and potential critical myelon damage to the patient if repositioned in the supine position during surgery.

### 2.4. Participants

Anaesthesiologists with different levels of training were enrolled. All participants had previous experience with TT and SGA exchange with VL or flexible endoscopy, with a usage of between 5 and 25 times prior to the study. All participants obtained a standardised briefing for each scenario. Furthermore, all participants were familiar with the manikin due to previous training, and no assistance was given during the scenarios by the instructors.

### 2.5. Measurements and Endpoints

The primary endpoint was a successful tracheal airway exchange within ten minutes after the start of the scenario and before severe hypoxemia (SpO_2_ < 80%) arose. Successful placement of the TT was confirmed by researchers with the ability to ventilate the lungs of the simulator or until the tip of the TT disappeared between the vocal cords, visualised by the applied video device. All scenarios started with an oxygen saturation of 95% and continuously decreased to 79% within ten minutes if the exchange of the TT or SGA failed within this defined time. Observation was limited to that time. After 10 min, for educational and psychological reasons, the scenario was continued, and proactive help was offered. To avoid bias, all participants performed airway management alone, and the participants were not allowed to watch each other. A successful tracheal intubation attempt was defined as tracheal tube placement in the trachea with a single blade insertion without manipulation by another provider or removal of the blade [31].

The secondary endpoints were the ‘time to successful airway exchange’, measured with a stopwatch (from the beginning of the scenario until successful airway exchange), and the following factors that are essential for airway exchange procedures and were recorded as dichotomous criteria (yes/no) [32]: ‘perception of the airway problem’, defined by the direct articulation of the problem by the participant; ‘performing preoxygenation’, defined by a rapid increase in the fraction of the inspiration of oxygen; ‘team communication’, defined as a global factor based on the items of situational awareness, problem identification, decision-making, workload distribution, and time management [33,34,35]; ‘optimising depth of anaesthesia’, defined by applying appropriate drugs; and ‘loss of the airway’, defined as cases where the participant removed the existing airway device without glottic visualisation or maintenance of the airway by an AEC. Based on this primary endpoint, we also observed the airway devices/instruments or airway techniques that were used to perform successful airway exchange.

The following demographics items were recorded: expertise in ‘years of anaesthesia experience’ and ‘anaesthesia experience’, where trainees were defined as having <5 years of anaesthesia experience and consultants had ≥5 years; a self-estimated number of performed SGA and TT exchanges.

### 2.6. Statistical Analysis

As there were previously no published data before the start of this study, this study essentially had exploratory intentions; therefore, all *p*-values should be interpreted descriptively. The sample size was determined by practical considerations. The available sample size of *n* = 30 used for the LTS and CUFF scenarios allowed a 95% confidence interval for the probability of success to be obtained, with the half-width not exceeding 0.19 (=19%) with a probability of at least 95%. If *n* = 60, as planned for the PRONE scenario, a 95% confidence interval for the probability of success with the half-width not exceeding 0.14 (=14%) can be obtained with at least 95% probability.

The data were compiled using a standard spreadsheet application (Excel 2016, Microsoft^®^, Redmond, WA, USA) and analysed with SAS 9.4, R 4.3.1, and GraphPad Prism (Version. 8.0 for MAC; GraphPad Software, La Jolla, CA, USA).

The success rates of the particular scenarios were displayed as percentages of all attempts of one scenario with the exact 95% confidence intervals, and the time to success was displayed by cumulative percentages of success over time. The demography and continuous and categorial variables were described by frequency tables and by the median (minimum (min), 25st quartile, 75th quartile, maximum (max)). Visualisation of the sequence of airway instruments applied throughout the scenarios was performed using river plots to gain traceability to a final endpoint: success (yes/no).

The categorical covariates ‘perception of the airway problem’, ‘performing preoxygenation’, ‘team communication’, ‘optimising depth of anaesthesia’, ‘loss of airway’, ‘anaesthesia experience’, ‘experience of SGA exchange’, and ‘experience of TT exchange’ were tested for associations with success using Fisher’s exact test. The continuous variable ‘years of experience’ was compared between the successful and unsuccessful outcomes using the Wilcoxon–Mann–Whitney test. Factors were considered associated with success if the *p*-value was less than 0.05.

## 3. Results

The demographics of the participants are displayed in Table 1. For the LTS (*n* = 30) and CUFF (*n* = 30) scenarios, observations could be completed. For PRONE (*n* = 59), *n* = 1 was discontinued due to technical problems; see Figure 1. The baseline characteristics of the participants were comparable between the groups (Table 1).

Table 2 shows the successful airway exchange rates and duration of intubation data for both groups. There were no differences in the successful exchange rate between the LTS group (27/30; 90%, 95% CI 73–98%) and the CUFF group (29/30; 97%, 95% CI 82.8–99.9%; *p* = 0.06), or in the first-attempt success rate between the LTS group 11/27 (41%) and the CUFF group (6/29; 21%; *p* = 0.14) within 10 min.

In the LTS group, 19/27 (70%) of the participants performed a successful airway exchange using VL with a Macintosh Blade, 3/27 (11%) used VL with a hyperangulated blade, 1/27 (4%) used DL, 1/27 (4%) used a Cook^®^ AEC, 1/27 (4%) used an FO, and 2/27 (7%) used a cricothyrotomy. In the CUFF group, 11/29 (38%) of the participants used VL with a hyperangulated blade, 9/29 (31%) used a Cook^®^ AEC, 5/29 (17%) used VL with a Macintosh blade, 1/29 (3%) used DL, 1/29 (3%) used an Aintree AEC, 1/29 (3%) used an S-guide AEC, and 1/29 (3%) participants used a cricothyrotomy for successful airway exchange. Table S1 (supplementary material) showing factors influenced on success in the particular scenarios. 

**Figure 2:**
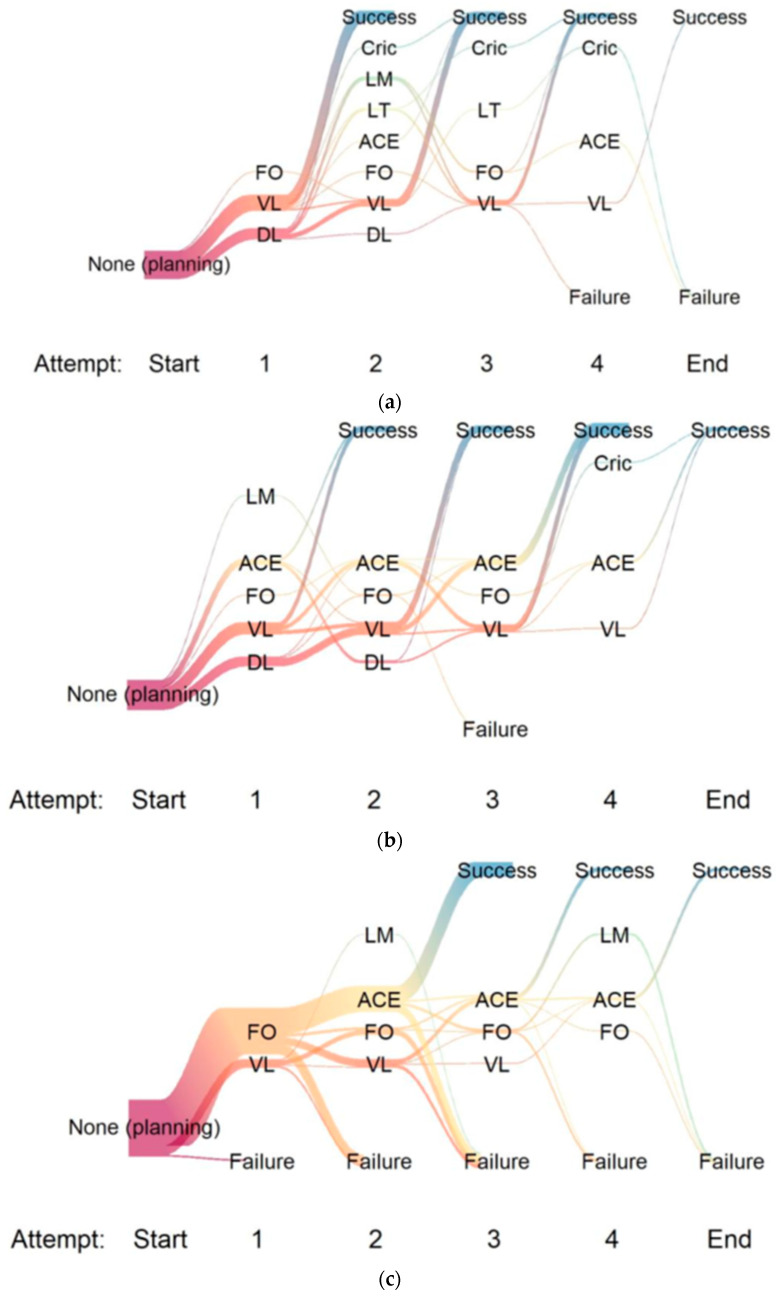
(**a**) LTS: River plot visualising the path to success or failure in the three scenarios during the particular attempts. (**b**) CUFF: River plot visualising the path to success or failure in the three scenarios during the particular attempts. (**c**) PRONE: River plot visualising the path to success or failure in the three scenarios during the particular attempts. Legend (Figure 2a–c): The river plots illustrate the paths of the applied instruments to success or failure of a scenario. The river plots generally start with a phase of planning, where no action was observed (Start: None (planning)). Then, the attempts were launched consecutively, coded by the number of the attempt (1, 2, 3, 4), and the following instruments were applied: LM: laryngeal mask, Stylet: any kind of AEC, FO: flexible endoscopy, VL: video laryngoscopy, DL: direct laryngoscopy. The thickness of the “rivers” is proportional to the number of participants using the particular instrument.

In the PRONE scenario, successful airway exchange within 10 min was achieved by 22/59 participants (37%, 95% CI 25.0–50.9%). Of those 22 successful participants, 17/22 (77%) used a Cook AEC, and 5/22 (23%) used a Muallem AEC. None of the participants were successful in the first attempt. A total of 15/22 (68%) needed two attempts, 4/22 (18%) needed three attempts, and 3/22 (14%) needed four attempts. The scenario was successfully completed in 8 min (IQR 6–8 (range of 6–10)). The pathways to success or failure are visualised in Figure 2a–c.

The explorative test concerning factors are as follows.

In the PRONE scenario, the factor ‘loss of the airway’ was associated with the failure of the scenario; the 22/59 (37%) participants that were successful within 10 min maintained the airway. Of the remaining unsuccessful 37 participants, 29/37 (76%) maintained the airway but did not manage the scenario within 10 min. The other 9/37 participants (24%) could not maintain a successful airway exchange in the complete scenario time of 20 min. In the LTS and CUFF groups, the factor ‘loss of the airway’ was not associated with success (*p* = 1.00 and 0.37).

All participants perceived the particular airway problem. None of the following factors influenced success within 10 min: ‘performing preoxygenation’, ‘team communication’, ‘optimising depth of anaesthesia’, ‘anaesthesia experience’, ‘experience of SGA exchange’, ‘experience of TT exchange’, ‘level of experience’, ‘years of experience’.

## 4. Discussion

This monocentric, prospective randomised clinical trial compared different airway techniques for the exchange of an existing airway in two simulation-based scenarios. To the best of our knowledge, no studies have described different airway techniques for the exchange of an SGA or TT before. This study shows that the exchange of a pre-existing SGA or TT is a time-critical procedure with an incidence of life-threatening effects. This similarly applies for trainees and consultant anaesthesiologists. Both groups showed a similar overall success rate for the airway exchange within 10 min with a low first-attempt success rate and the need for multiple attempts. In the additional PRONE scenario, we detected a single factor (‘loss of the airway maintenance’) that presented a noticeable association with success under 10 min. All participants that were successful did not lose the airway but maintained it until the final exchange of the TT. This fact advocates for thorough planning and deliberate evaluation before any manipulation of any pre-existing applied airway device is performed. Especially in fixed prone positioning, the maintenance of any pre-existing airway by an AEC is crucial.

### 4.1. Exchange of the LTS

Anaesthesiologists successfully managed the exchange of an LTS in under 10 min in 90% of cases, mainly by using VL with a Macintosh blade, and there was a need for a maximum of four attempts to complete the scenario within 10 min. The use of the LTS is more frequent by German paramedics or non-anaesthesiologists compared to anaesthesiologists, and after hospital admission, the LTS usually needs to be exchanged for a TT for further mechanical ventilation. The proportion of prehospital airway management using an LTS is cited as being as high as 9.3% (14 of 150 non-trauma critically ill patients) and 9% (48 of 538 patients admitted to an emergency department) [4,6]. The research on comparable data is challenging due to the diversity of the study designs, in addition to governmental airway management registries that are not accessible to the public.

In the present study, for the exchange of a pre-existing LTS, the participants successfully used VL with a Macintosh blade in 71% of cases, a hyperangulated blade in 11%, and DL in 4%. These are the most frequently applied airway devices in the current clinical routine. Airway techniques reported in 56 trauma and 48 emergency patients for definitive airway management after LTS insertion include tracheostomy (36% and 29%), DL (18% and 21%), VL (15% and 21%), FO (20% and 6%), and AEC (11% and 22%) [1,6].

The evaluation of the airway condition may be performed quickly through routine application. In comparison to the previous data, we obtained a remarkably higher ratio of VL and only one (4%) application of AEC in this scenario. Maintaining continuous access during the exchange of an existing airway via an AEC can be an essential component of an airway strategy in high-risk patients. The data of previous studies were collected from 2009 to 2016 [1], 2007 to 2012 [6], 2010 to 2015 [7], and in the present study in 2019. During this time, VL increasingly developed to a commonly applied technique [17]. Anaesthesiologists proficient in VL should use the technique they are mostly familiar with. Schalk et al. demonstrated, in 19/20 patients (95%), a successful video-based exchange of a pre-existing LTS performed by consultant anaesthesiologists at a tertiary hospital in Germany [31].

In another study of 453 trauma patients presenting with an LTS, intubation was performed using VL with an LTS in situ (i.e., the LTS was in the patient’s oropharynx during VL) in 48.2% of cases, with VL after the removal of an LTS in 25.3%, direct laryngoscopy after the removal of an LTS in 24.9%, and further techniques used [7]. The authors recommend leaving the LTS in place during intubation using VL and maintaining the airway with a bougie/AEC. Similarly, case reports recommend using VL with a bougie [8,12]. This technique enables the maintenance of oxygenation by the LTS and results in less tissue trauma because the LTS is kept in place until the TT is completed, and a bougie facilitates the maintenance of the airway. One case report described an exchange technique using flexible endoscopy and a bougie in the rare situation of a kinked LTS [15]. However, the application of an AEC in addition to VL prevents the loss of the airway. This may happen due to complications described for LTS, like tongue engorgement, glottic oedema, or other adverse events [6]. Genzwuerker et al. demonstrated, in their study, a first-attempt success rate of 80% by using an FO with an AEC for the successful exchange of a first-generation laryngeal tube within 3 min [31]. Therefore, an AEC facilitates maximum airway control. So, there is a high variety of techniques that should be applied depending on the provider, equipment, and situation.

This scenario does not present a standard situation for anaesthesiologists. The essence of a “Plan A” is recommended in different airway guidelines and to increase successful intubation in the first attempt or to minimise the likelihood of multiple attempts [16,17,18]. The authors described different airway techniques, e.g., VL, which enables a better glottic view. In this scenario, the relationship between choices and decision making (in the exchange of an LTS) is a complex one. The term “choice overload” is used in other areas to describe situations where an increase choice can result in delayed decision-making or the inability to decide at all [36]. By using decision-making tools that focus on the problem at hand, anaesthesiologists can quickly make decisions based on limited information or options, producing results that are comparable to those made by complex models with multiple variables. This not only has technical benefits, but also helps the airway team become more familiar with the equipment required for emergency situations. Furthermore, competence in using the equipment required for managing unexpected airway difficulties is an expected minimum for a prepared airway practitioner, and expertise is the goal.

### 4.2. Exchange of the Tracheal Tube with Cuff Leakage

For an airway exchange of a TT based on cuff leakage, the success rate was 97% using VL and AEC in similar proportions (VL: 38% and 31%). We found techniques to bridge the TT by technical applications during ongoing surgery [33]. There are no data about incidences or trials concerning the exchange of a TT with cuff leakage during general anaesthesia or during ventilation in critical care patients. In a review, authors suggested the use of AEC, describing several techniques and pitfalls [3]. A case report cited complications, like severe pneumothorax [34]. The scenarios of TT cuff defects represent a simple problem, where 97% of the participants were overall successful but the first-attempt success rate was only 21%. Nevertheless, 38% of the participants accomplished the scenario using VL, and 31% did so using a Cook ACE or 4% an Aintree AEC. This can probably be explained by the high level of experience with VL by anaesthesiologists. Furthermore, severe complications caused by an AEC may be lessened with the use of VL in the hands of an experienced anaesthesiologist [10], facilitating sufficient airway control. This is especially crucial in intensive care patients. They often present a change in airway conditions due to pathophysiological oedema caused by, e.g., a capillary leak in severe sepsis. Even in the case of a normal airway evaluated under pre-intensive conditions, difficult airways are frequent under intensive medicine situations [17,35]. Thus, the application of AECs is probably most recommendable for a TT airway exchange under intensive conditions. The National Audit Project 4 illustrated that 47% of anaesthesia-related events are associated with primary airway problems, and failed intubation was the most frequently recorded event [35]. The blind insertion of an AEC as an aid to exchange a TT can be a very effective tool, but soft tissue trauma can result; therefore, undue pressure should be avoided. Therefore, the authors recommend that airway exchange catheters should be used only according to their manufacturers’ instructions. This includes limiting the depth of insertion (<26 cm from the distal end), and their use with a high-pressure source for ventilation should be reserved for special circumstances of necessity and requires the highest standards.

### 4.3. Fixated Prone Positioning

Thirty-seven percent of the participating anaesthesiologists managed the scenario in the prone position successfully within 10 min, mostly using AECs.

The prone position of patients during anaesthesia is required to provide operative access for a wide variety of surgical emergencies as elective procedures. Airway management in this position is a challenge for the anaesthetist because it creates obstacles that impair the ability to achieve endotracheal intubation by direct laryngoscopy. Airway complications in a fixed prone position are mostly life-threatening situations. Recent recommendations have been published regarding situations in which the airway is lost entirely by an accidental extubation [5,24]. The applications of SGA, VL, and FO have been discussed, controversially [2,25,26,37,38,39,40,41,42,43]. However, we found no publications concerning the complications of a correctly placed TT when undergoing surgery in prone positioning in which the airway maintenance was not entirely lost.

With a rapid change in desaturation or decreased ventilation, primary equipment issues have to be ruled out quickly. Case reports, for instance, have cited obstruction by a cuff overinflation [20,34]. In the present study, an incomplete but severe and irreversible obstruction by tracheal secretion was simulated. The primary focus should be to assure the correct TT positioning before an airway exchange attempt [39]. This was performed by 82% of the participants using an FO and by 15% using VL in the first attempt. There was no success in the first attempt because the participants first evaluated that the TT was in the trachea without dislocation.

If the TT is in the trachea and has to be exchanged, the airway should be maintained. This can be assured using an AEC so that the airway remains splinted [3]. The factor ‘loss of the airway’ in the present study was one of the most important factors for failure in the fixed prone position scenario.

### 4.4. Factors Influencing Success

As cited above, the factor ‘loss of the airway’ was the most crucial factor associated with failure in the prone position; however, this was not the case in the other scenarios.

Our sample consisted of anaesthesiologists presenting a median of seven years of professional experience. However, the factor ‘anaesthesia experience’ and the self-reported experience of exchange procedures (LTS or TT) did not influence success in any scenario. Although cricothyrotomy was not a major issue in the present study, previous simulation-based airway studies used this skill to evaluate the skills of physicians [42]. One study found that inexperienced emergency medicine residents (1st year) needed longer for cricothyrotomy in an emergency scenario than more experienced residents (2nd and 3rd year) [44,45]. Another study found that younger physicians managed cricothyrotomy scenarios faster and better than older anaesthesiologists, where the number of years of age (mean of 37 vs. mean of 58) was far out the range of the present study in years of experience [40]. Experience itself may not be the crucial advantage. We found no studies providing evidence that experience is a factor influencing success in airway management, nor did the present study. Competence in procedures and skills may be less a matter of anaesthesia experience or time spent gathering experience, and more so a matter of training, physical capability, and attitude [46,47,48].

## 5. Limitations

Simulation-based research always suffers from a lack of haptic feedback and clinical signs of a real patient, and the results are difficult to translate directly into patient care [27,28]. This is a general concern and has to be taken into account for any interpretation. It is demanding to deduce recommendations for the clinical reality from such studies. Nevertheless, we decided to perform a simulation-based research study due to the potential to constitute standardised situations and avoid crucial harm to specific patients. A controlled trial concerning the three situations is nearly impossible in clinical reality. Here, simulation-based research provided the opportunity to collect procedural data without the stressful and dynamic conditions of patient care. Generalisability in terms of the validity and applicability of our results is limited to experienced airway providers. Our sample held a median of 7 years of professional experience. The data collected account for consultants caring for patients under the three particular conditions defined. This study does not provide data to state any recommendations for inexperienced providers concerning these situations.

One limitation of the present study is the fact that we did not perform a sample size or power calculation. On the one hand, to our knowledge, there are no data facilitating such a calculation for the situations we explored, so we could not reliably provide a power analysis. On the other hand, we obtained as many participants as we could recruit within the available time. Thus, the sample size was limited by time, personal resources, and a duty roster. A sample size and power calculation for future studies can be based on the data of the present study.

## 6. Conclusions

If there is any airway device already applied in a patient providing any kind of sufficient oxygenation, the results of the present study support a deliberate evaluation of the present airway condition and foster thorough planning for exchange strategies. This should be considered before any manipulation on the pre-existing device is made. Spontaneous or undeliberate actions must be avoided. If there is any condition of a potential difficult airway per se, the airway should be maintained in any case, for example, by an airway catheter.

## Figures and Tables

**Table 1:** Participant characteristics; airway and anaesthetists characteristics presented as numbers (proportion).

	LTS Group *n* = 30	CUFF Group *n* = 30	*p*-Value
Level of training			
trainees	8 (27%)	8 (27%)	
consultants	22 (73%)	22 (73%)	0.0007
Experience of airway exchange (SGA)			
<5 times	14 (47%)	14 (47%)	0.99
5–10 times	8 (27%)	7 (23%)	
11–15 times	4 (13%)	5 (17%)	
16–25 times	4 (13%)	4 (13%)	
Experience of airway exchange (TT):			
<5 times	15 (50%)	18 (61%)	0.6
5–10 times	7 (23%)	7 (23%)	
11–15 times	6 (20%)	4 (13%)	
16–25 times	2 (6%)	1 (3%)	

Abbreviations: SGA: Supraglottic airway device; TT: Tracheal tube.

**Table 2:** Outcome parameters presented as number (proportion) or median (25th- 75th percentile) [range]. ^a^ Wilcoxon Test. ^b^ Fisher’s Exact Test.

	LTS (*n* = 30)	CUFF (*n* = 30)	*p*-Value
No. of attempts			
First attempt	11 (41%)	6 (21%)	0.14 ^b^
Second attempt	9 (33%)	7 (24%)	0.76 ^b^
Third attempt	6 (22%)	12 (41%)	0.15 ^b^
Fourth attempt	1 (4%)	4 (14%)	0.35 ^b^
Overall success rate	27(90%)	29/30 (97%)	0.06 ^b^
Intubation time (min)	6 (5–7 [3–9])	7 (6–8 [4–10])	0.15 ^a^

## Data Availability

Data is unavailable due to privacy or ethical restrictions.

## Supplementary Materials

The following supporting information can be downloaded at: https://www.mdpi.com/article/10.3390/jcm13010016/s1, Table S1: Factors that were tested (Fisher’s exact test: f, Wilcoxon–Mann–Whitney test: m) concerning their influence on success in the particular scenarios.

## References

[1] Hernandez M.C., Aho J.M., Zielinski M.D., Zietlow S.P., Kim B.D., Morris D.S. (2018). Definitive airway management after pre-hospital supraglottic airway insertion: Outcomes and a management algorithm for trauma patients. Am. J. Emerg. Med..

[2] Abrishami A., Zilberman P., Chung F. (2010). Brief review: Airway rescue with insertion of laryngeal mask airway devices with patients in the prone position. Can. J. Anaesth..

[3] Udomtecha D. (2012). Airway tube exchanger techniques in morbidly obese patients. Anesthesiol. Res. Prac..

[4] Bernhard M., Bax S.N., Hartwig T., Yahiaoui-Doktor M., Petros S., Bercker S., Ramshorn-Zimmer A., Gries A. (2019). Airway Management in the Emergency Department (The OcEAN-Study)—A prospective single centre observational cohort study. Scand. J. Trauma. Resusc. Emerg. Med..

[5] Bosch L., Pacreu S., Castelltort L., Gallart L. (2021). Accidental extubation in prone position: Report of two cases and proposal of an algorithm for airway management. Eur. J. Anaesthesiol..

[6] Subramanian A., Garcia-Marcinkiewicz A.G., Brown D.R., Brown M.J., Diedrich D.A. (2016). Definitive airway management of patients presenting with a pre-hospital inserted King LT(S)-D™ laryngeal tube airway: A historical cohort study. Can. J. Anaesth..

[7] Dodd K.W., Klein L.R., Kornas R.L., Driver B.E., Ho J.D., Reardon R.F. (2016). Definitive airway management in emergency department patients with a King laryngeal tube™ in place: A simple and safe approach. Can. J. Anaesth..

[8] Klein L., Paetow G., Kornas R., Reardon R. (2016). Technique for Exchanging the King Laryngeal Tube for an Endotracheal Tube. Acad. Emerg. Med..

[9] Monclus E., Garcés A., Artés D., Mabrock M. (2008). Oral to nasal tube exchange under fibroscopic view: A new technique for nasal intubation in a predicted difficult airway. Pediatr. Anesth..

[10] Mort T.C., Braffett B.H. (2015). Conventional Versus Video Laryngoscopy for Tracheal Tube Exchange: Glottic Visualization, Success Rates, Complications, and Rescue Alternatives in the High-Risk Difficult Airway Patient. Anesth. Analg..

[11] Raveendran R., Sastry S.G., Wong D.T. (2013). Tracheal extubation with a laryngeal mask airway and exchange catheter in a patient with a difficult airway. Can. J. Anaesth..

[12] Sandefur B.J., Driver B.E., Brown C.A., Reardon R. (2020). F. Definitive Airway Management of Patients with a King Laryngeal Tube in Place in the COVID-19 Pandemic. West. J. Emerg. Med..

[13] Gaither J.B., Matheson J., Eberhardt A., Colwell C.B. (2010). Tongue engorgement associated with prolonged use of the King-LT laryngeal tube device. Ann. Emerg. Med..

[14] Mort T.C. (2007). Continuous airway access for the difficult extubation: The efficacy of the airway exchange catheter. Anesth. Analg..

[15] Kelm D.J., Zhu X., Diedrich D.A., Ritter M.J., Gajic O. (2018). Fiberoptic-Guided Use of an Airway Exchange Catheter During Exchange of a Kinked King LTS-D Laryngeal Tube for an Endotracheal Tube: A Case Report. A&A Prac..

[16] Frerk C., Mitchell V., McNarry A., Mendonca C., Bhagrath R., Patel A., O’sullivan E., Woodall N., Ahmad I. (2015). Difficult Airway Society 2015 guidelines for management of unanticipated difficult intubation in adults. Br. J. Anaesth..

[17] Higgs A., McGrath B., Goddard C., Rangasami J., Suntharalingam G., Gale R., Cook T. (2018). Guidelines for the management of tracheal intubation in critically ill adults. Br. J. Anaesth..

[18] Apfelbaum J.L., Hagberg C.A., Connis R.T., Abdelmalak B.B., Agarkar M., Dutton R.P., Fiadjoe J.E., Greif R., Klock P.A., Mercier D. (2022). 2022 American Society of Anesthesiologists Practice Guidelines for Management of the Difficult Airway. Anesthesiology.

[19] Mort T.C. (2009). Tracheal tube exchange: feasibility of continuous glottic viewing with advanced laryngoscopy assistance. Anesth. Analg..

[20] Johnson K.M., Lehman R.E. (2012). Acute management of the obstructed endotracheal tube. Respir. Care.

[21] Saunders T.G., Gibbins M.L., Seller C.A., Kelly F.E., Cook T.M. (2019). Videolaryngoscope-assisted flexible intubation tracheal tube exchange in a patient with a difficult airway. Anaesth. Rep..

[22] Barker J., Koeckerling D., West R. (2020). A need for prone position CPR guidance for intubated and non-intubated patients during the COVID-19 pandemic. Resuscitation.

[23] Edgcombe H., Carter K., Yarrow S. (2008). Anaesthesia in the prone position. Br. J. Anaesth..

[24] Gaszynski T. (2020). Algorithm for management of sudden unexpected extubation in patient positioned in prone position. Anaesthesiol. Intensiv. Ther..

[25] Hung M.-H., Fan S.-Z., Lin C.-P., Hsu Y.-C., Shih P.-Y., Lee T.-S. (2008). Emergency airway management with fiberoptic intubation in the prone position with a fixed flexed neck. Anesth Analg..

[26] De Cosmo G., Congedo E. (2017). Unintentional tracheal extubation during prone position: What is the best rescue airway device?. J. Emerg. Trauma. Shock.

[27] Schnittker R., Marshall S., Horberry T., Young K.L. (2018). Human factors enablers and barriers for successful airway management—An in-depth interview study. Anaesthesia.

[28] Ott T., Stracke J., Sellin S., Kriege M., Toenges G., Lott C., Kuhn S., Engelhard K. (2019). Impact of cardiopulmonary resuscitation on a cannot intubate, cannot oxygenate condition: A randomised crossover simulation research study of the interaction between two algorithms. BMJ Open.

[29] Cheng A., Kessler D., Mackinnon R., Chang T.P., Nadkarni V.M., Hunt E.A., Duval-Arnould J., Lin Y., Cook D.A., Pusic M. (2016). Reporting guidelines for health care simulation research: Extensions to the CONSORT and STROBE statements. Adv. Simul..

[30] Schalk R., Weber C.F., Byhahn C., Reyher C., Stay D., Zacharowski K., Meininger D. (2012). Reintubation using the C-MAC videolaryngoscope. Implementation in patients with difficult airways initially managed with in situ laryngeal tubes. Anaesthesist.

[31] Lee R.A., VAN Zundert A.A.J., Maassen R.L.J.G., Wieringa P.A. (2012). Forces applied to the maxillary incisors by video laryngoscopes and the Macintosh laryngoscope. Acta Anaesthesiol. Scand..

[32] Genzwuerker H.V., Vollmer T., Ellinger K. (2002). Fibreoptic tracheal intubation after placement of the laryngeal tube. Br. J. Anaesth..

[33] Baker P. (2015). Preparedness and education in airway management. Anesthesiol. Clin..

[34] El-Orbany M., Salem M.R. (2013). Endotracheal tube cuff leaks: Causes, consequences, and management. Anesth. Analg..

[35] Harris K., Chalhoub M., Maroun R., Elsayegh D. (2012). Endotracheal tube exchangers: Should we look for safer alternatives?. Heart Lung.

[36] Cook T.M., Woodall N., Frerk C. (2011). Fourth National Audit Project. Br. J. Anaesth..

[37] Jessup R.K., Ritchie L.E., Homer J. (2020). Hurry up and decide: Empirical tests of the choice overload effect using cognitive process models. Decision.

[38] Frutos-Vivar F., Ferguson N.D., Esteban A., Epstein S.K., Arabi Y., Apezteguía C., González M., Hill N.S., Nava S., D’empaire G. (2006). Risk factors for extubation failure in patients following a successful spontaneous breathing trial. Chest.

[39] Hofstetter C., Scheller B., Hoegl S., Mack M.G., Zwissler B., Byhahn C. (2010). Cuff overinflation and endotracheal tube obstruction: Case report and experimental study. Scand. J. Trauma Resusc. Emerg. Med..

[40] Ilgen J.S., Ma I.W.Y., Hatala R., Cook D.A. (2015). A systematic review of validity evidence for checklists versus global rating scales in simulation-based assessment. Med. Educ..

[41] Davies J.M. (2005). Team communication in the operating room. Acta Anaesthesiol. Scand..

[42] Oshika H., Koyama Y., Taguri M., Maruyama K., Hirabayashi G., Yamada S.M., Kohno M., Andoh T. (2018). Supraglottic airway device versus a channeled or non-channeled blade-type videolaryngoscope for accidental extubation in the prone position: A randomized crossover manikin study. Medicine.

[43] Thiel D., Houten J., Wecksell M. (2014). Accidental tracheal extubation of a patient in the prone position. A & A Case Rep..

[44] Salem M.R. (2001). Verification of endotracheal tube position. Anesthesiol. Clin. N. Am..

[45] Siu L.W., Boet S., Borges B.C.R., Bruppacher H.R., LeBlanc V., Naik V.N., Riem N., Chandra D.B., Joo H.S. (2010). High-fidelity simulation demonstrates the influence of anesthesiologists’ age and years from residency on emergency cricothyroidotomy skills. Anesth. Analg..

[46] Girzadas D.V., Clay L., Caris J., Rzechula K., Harwood R. (2007). High fidelity simulation can discriminate between novice and experienced residents when assessing competency in patient care. Med. Teach..

[47] Lingard L., Regehr G., Espin S., Whyte S. (2006). A theory-based instrument to evaluate team communication in the operating room: Balancing measurement authenticity and reliability. Qual. Saf..

[48] Butchibabu A., Sparano-Huiban C., Sonenberg L., Shah J. (2016). Implicit Coordination Strategies for Effective Team Communication. Hum. Factors.

